# Shirt sponsorship by gambling companies in the English and Scottish Premier Leagues: global reach and public health concerns

**DOI:** 10.1080/14660970.2018.1425682

**Published:** 2018-01-17

**Authors:** Christopher Bunn, Robin Ireland, Jonathan Minton, Daniel Holman, Matthew Philpott, Stephanie Chambers

**Affiliations:** a College of Social Sciences, Institute of Health and Wellbeing, University of Glasgow, Glasgow, UK; b European Healthy Stadia Network, Liverpool, UK; c College of Social Sciences, Urban Studies, University of Glasgow, Glasgow, UK; d Department for Sociological Studies, University of Sheffield, Sheffield, UK; e Social and Public Health Sciences Unit, Medical Research Council/Chief Scientist Office, University of Glasgow, Glasgow, UK

**Keywords:** Gambling, sponsorship, football (soccer), problem gambling, public health

## Abstract

While the nature of gambling practices is contested, a strong evidence base demonstrates that gambling can become a serious disorder and have a range of detrimental effects for individuals, communities and societies. Over the last decade, football in the UK has become visibly entwined with gambling marketing. To explore this apparent trend, we tracked shirt sponsors in both the English and Scottish Premier Leagues since 1992 and found a pronounced increase in the presence of sponsorship by gambling companies. This increase occurred at the same time the Gambling Act 2005, which liberalized rules, was introduced. We argue that current levels of gambling sponsorship in UK football, and the global visibility it provides to gambling brands, is a public health concern that needs to be debated and addressed. We recommend that legislators revisit the relationship between football in the UK and the sponsorship it receives from the gambling industry.

## Introduction

In the UK, the social practice of gambling has been subject to varying degrees of legislative control, reaching what some have seen as a ‘high water mark’ in 1906.^1^ From the 1906 Street Betting Act to the 1960 Betting and Gaming Act, gambling was essentially an underground activity and one subject to significant (if evadable) punishment. The 1960 legislation set a new direction that saw greater toleration of gambling in Britain, and introduced high street betting shops. Between 1960 and the advent of the National Lottery Act in 1993, gambling largely operated on the principle of ‘unstimulated demand’. However, in 2005, the New Labour administration swept this paternalism away, via the 2005 Gambling Act, which significantly liberalized gambling laws, endorsed (through regulation) online gambling and permitted gambling companies to run television adverts. In an era of globalization, these policy shifts in the UK have broader consequences than those traditionally associated with the territorially bound governance of states, and have contributed to the ‘convergence of a global passion for sport and a global passion for gambling’.^2^ This convergence has received insufficient attention in the public health literature, especially in the UK, and is particularly visible in football contexts.

Amongst the most visible partnerships that gambling companies have entered into in the UK since the 2005 Act, have been with clubs in the English Premier League (EPL). The EPL is an important export for the UK economy with broadcasters paying subscriptions to the EPL for the right to show matches and highlights across 225 territories. The EPL claims that during the 2013/2014 season, 2.3 billion fans were engaged in the competition for the full 10 months of its duration and in the 2014/2015 season, that it reached an audience of 3 billion in-home viewers.^3^ The current television deal saw Sky Sports and BT Sport pay £5.136 billion for broadcast rights between 2016 and 2019.^4^ It is clear, therefore, that the EPL can be a significant global marketing vehicle for any industry, whether through shirt sponsorship, advertising hoardings, league sponsorship or stadium sponsorship. In recent years, the gambling industry has made use of this marketing vehicle through a range of sponsorship deals with EPL clubs. The industry has also made significant inroads within the wider English Football League, with one gambling company sponsoring the second, third and fourth-tier leagues in England.

In contrast to this, the Scottish ‘Premier League’ (SPL)^5^ receives less global attention and less income from selling its TV rights. While Scottish football enjoys amongst the highest per-capita match day attendance in Europe, its global TV reach is less than half the number of territories covered by the EPL.^6^ The current SPL television deal reflects the disparity of reach between the EPL and SPL, and saw a combined bid from Sky Sports and ESPN raise just £80 million for the right to broadcast 30 matches a year between 2014 and 2017.^7^ However, the lower global reach and apparent value of the television rights for Scottish football has not rendered it immune to the interests of gambling companies. In May 2015, the Scottish Professional Football League entered into a sponsorship agreement with two gambling organizations: one put its name to the full set of 4 professional leagues; and the other to the Scottish league cup.

In this paper we aim to document the number of gambling companies that have sponsored the shirts of clubs who have played in the EPL and SPL and to consider whether the 2005 Gambling Act is associated with any change in shirt sponsorship patterns. We contextualize our analysis with literature on gambling and explore how this fits with other research on gambling and sport. As the debate on gambling and sport in the UK is an emerging one, we take an interdisciplinary public health perspective that considers the potentially global impacts of recent legislative and cultural shifts.

## Conceptualizing gambling and its social impacts

In public health, the predominant conceptualization of gambling has come from researchers in psychiatry and related biomedical disciplines. In 1980, the third edition of the Diagnostic and Statistical Manual of Mental Disorders (DSM) introduced ‘compulsive gambling’ into its inventory of mental health conditions.^8^ The fourth edition of the DSM shifted away from labelling gambling as a ‘compulsion’ and replaced it with ‘pathological gambling’. Finally in the more recent DSM V, terminology shifted again to ‘gambling disorder’ (GD). To be diagnosed with GD, a person must exhibit 4 of 9 traits, such as ‘often gambling when feeling distressed’ or ‘lying to conceal gambling activity’.^9^ In the absence of conclusive biopsychological evidence, GD is most commonly positioned as a non-substance related form of substance abuse disorder.^10^ These psychiatric and neurobiological approaches locate gambling in a diseased brain and tend to offer behavioural solutions, such as CBT, or (in more extreme cases) pharmaceutical solutions such as opioid antagonists, serotonin reuptake inhibitors (SRIs) and mood stabilizers.^11^

Public health perspectives have generally adopted a broader view of gambling. In a review of the state of public health knowledge on gambling, Shaffer and Korn came to the conclusion that:

Prevalence research implicitly encourages a clinical, idiographic analysis of gambling disorders – as if understanding individual attributes offered a sufficient explanation. However, public health strategies historically have focused on the interactions among the host, agent, and environment – in this case the gambler, his or her psychological expectations, resistances, and vulnerabilities, and the *social setting* within which people gamble. This interactive perspective on gambling disorders encourages greater involvement from public health workers and a multilevel integrated approach to interventions. (Italicised emphasis added)^12^

This critique of the individualized accounts of gambling offered by biomedicine adopts the view that gambling is a socially situated form of action. Extending this perspective, Reith and Dobbie’s research has addressed the shortcomings of taking an exclusively psychiatric viewpoint on gambling. They note that gambling practices are also influenced by legislative and socio-economic contexts, local environment, family, friends and colleagues.^13^ In addition to these influences, Reith and Dobbie have also observed that gambling practices can shift and mutate and that gambling ‘careers’ ‘ebb and flow over time, influenced in myriad ways by the social environments which gamblers inhabit, as well as the personal characteristics they bring to games’.^14^

While many psychiatrists and neurobiologists locate gambling in the minds of diagnosable individuals, it is clear social environments play a highly influential (and complex) role in the production of disordered gamblers. Of these broader social environments, the legislative context seems to be particularly important. An analysis of gambling expenditure before and after the introduction of the UK National Lottery in 1994, following the 1993 National Lottery Act, demonstrated that the number of households spending 10% or more of their total income on gambling products rose fourfold from 0.4 to 1.7%. The shift was especially pronounced in lower income groups: the number of households earning less than £200 per week that spent 10% or more of total income on gambling rose from 0.6 to 3.2%.^15^ Since the introduction of the 2005 act, population-adjusted levels of disordered gambling rose from 0.6% (as measured in both 1999 and 2007) to 0.9% (2010).^16^

From a public health perspective, the outcomes of disordered gambling also go beyond individuals. Indeed, the consequence for gamblers’ social networks and the societies in which they live are concerning. In most cases, disordered gambling manifests in ways that erode autonomy and security, and damage the quality of inter-personal relationships.^17^ At a macro level, societies also incur costs in the domains of health care, welfare and employment, housing and criminal justice. A recent report from the Institute for Public Policy Research estimates that the burden disordered gambling places on the public purse in the UK could be as great as £1.16 billion per annum.^18^

In simple terms, psychiatrists and biomedical professionals often view disordered gambling as a mental illness, in the sense that it displays similar symptoms. However, public health and sociological perspectives argue that it cannot be isolated within the individual: social contexts matter. The consequences of disordered gambling also extend beyond the individual, to families, friends, colleagues and tax-payers. In this paper, we adopt an interdisciplinary public health approach to disordered gambling that views it in the broad terms set out by Shaffer and Corn; as an interaction between an embodied individual, his or her ways of perceiving and interpreting the world, and the social contexts in which he or she resides and acts.

## Gambling and sport

While literature on the relationship between sport and gambling is still emerging in the UK, it has received some significant attention from researchers in other countries. Academics in Australia have made particularly important contributions. In a 2006 study of sports sponsorship across the top eight sports for 5–17 year olds, researchers identified gambling as the most common sponsor, representing 18.8% of the sample of 107.^19^ Related to this, a content analysis of in-stadia advertising during round 12 of the Australian Football League Premiership 2011 season identified that, per match, a mean of 58.5 episodes of sports betting marketing occurred, totalling a mean of 341.1 min.^20^ Following on from this, a similar content analysis of television coverage of a three-game Rugby League series found 332 episodes of gambling marketing lasting a total of 26.16 min, over 360 min of coverage.^21^

The Australian literature has not just established the close links between gambling sponsorship and professional sport, it has also begun to explore the effects. A recent exploratory study suggested that the heavy presence of gambling sponsorship in an Australian rules football competition may have encouraged gambling intentions.^22^ In a study of adolescents, the intention to gamble once reaching the age of 18 was associated with participating in and watching televised sports.^23^ Another study, by Bestman and colleagues, has shown that children aged 5–12 are able to associate gambling brands with specific sports and sports clubs.^24^ Finally, a further study has looked at the affective responses to gambling adverts during televised sport, and proposed a model for interpreting the variety of responses which positions viewers’ demographic characteristics, viewing patterns, social setting and advertising modalities as moderators of message reception.^25^

Research literature on North American sport and betting, while less developed than the Australian literature, has also emerged in recent decades. In 2004, McKelvey noted that professional sport organizations in the United States and Canada were growing quickly, citing an increase from 22 teams sponsored by a gambling company to 48 between 1996 and 2002. In the American context, this increase occurred despite limited opportunities to gamble on sports events, with only 4 states (Nevada, Delaware, Oregon, Montana) legally allowed to license such wagers.^26^ An analysis of online betting patterns in the national hockey league and national basketball association has suggested that betting behaviour is close to that of fan behaviour, with bettors attracted to the better teams, televised games, uncertain outcomes, and games early in the season. The authors of this study (Paul and Weinbach) conclude that most bettors are not investors, but consumers whose betting complements their viewing and game attendance.^27^ Finally, a recent survey of 580 students from across the United States revealed that deregulation of sports gambling would make men, those who are motivated by economic incentives, and people measured to be greater sports fans, more likely to express an interest in gambling products.^28^

Literature relating to Europe, and the UK specifically, is in its infancy. A study of German sports betting which used an online survey to capture data on sports-betting behaviour suggested that ‘the typical sports-bettor is 32 years old and male, has a low household income, is highly interested in sports, and is willing to take risks’.^29^ Such data are not available for the UK, but the role of gambling in English football has received recent critical attention. Jones has noted that football in the UK has embraced gambling sponsorship. He points out that while current UK law prohibits gambling marketing on television before 9 pm, football coverage is exempt from this rule, whatever time of the day it is broadcast. Jones also observes that a significant number of professional footballers have had very public relationships with gambling and that this visibility plays a role in normalizing gambling.^30^

While the literature on Europe and the UK is still developing, an African perspective has emerged. The spread of satellite television technologies has taken coverage of European football into remote parts of Africa, where these leagues were already extremely popular. In Uganda, for example, the anthropologist Vokes notes that the arrival of televised coverage of the EPL in the village that is the focus of his fieldwork ‘has also created a new interest in gambling in the village.’^31^ More detailed work on this topic has been carried out in Nigeria. Akanle and Kolade have documented the football betting landscape in Nigeria, where there is little regulation and ‘Anyone with the most basic equipment like internet connections … can start [a] football betting business.’^32^ They report that of 300 Nigerian bettors surveyed, a majority were between 21–30 years old (54.7%), male (79%) and derive their betting stake from unemployment benefit (41%).^33^ From the same survey data, Akanle and Kolade also report that 31% placed a bet daily and 20% four times a week.^34^

Examining the literature to date, it is clear that gambling companies across the world have made and continue to make use of the popularity of sport to increase the visibility of their products. The relationship is deeper than this, however: gambling seems to have become entangled with the act of consuming sports, as Paul and Weinbach demonstrate for the NHL and NBA, and Jones suggests of the EPL. Bestman and colleagues claim that this entanglement is internalized by children, and Akanle and Kolade establish that it is having an impact in Nigeria. Taken together, this literature raises concerning questions about the role sports are playing in the normalization and perpetuation of gambling. In what follows, we contribute to the literature by looking at shirt sponsorship by gambling companies amongst teams who have played in the EPL or SPL.

## Methods

In this paper, we have taken an inclusive approach to football clubs included in our analysis of shirt sponsorship. Since 1992/1993 the EPL has hosted a total of 38 teams and since 1998/1999, 19 teams have played in the SPL. In both the EPL and SPL, the clubs that rise and fall through from the ‘premier league’ to lower divisions are still of interest to our exploration of shirt sponsorship. Many of these teams continue to enjoy large fan bases (both locally and via televised games), due to their historical prestige or ‘symbolic capital’.^35^ For example, Nottingham Forest have not played in the EPL since the 1998/1999 season, but retain a large following and have consistently attracted >20,000 fans to their league matches since relegation.^36^ Similarly, Hibernian F.C., one of the two major clubs in Edinburgh, until 2017 had not played in the SPL since the 2013/2014 season, yet retained comparatively high attendance rates of between 9 and 15,000 fans.^37^

We consulted the websites of the EPL and SPL to identify the teams that have participated in these leagues since their inaugural seasons. All teams who have played at least one season in the EPL or SPL were included in our analysis to establish an EPL group (EPLG) and an SPL group (SPLG). We then used a combination of the official league handbooks from each season (where available) and an online football shirt archive site, www.historicalfootballkits.com, to identify shirt sponsors for each of these teams. Images of shirts were viewed and shirt sponsors were entered into a database, with the teams as cases and the seasons as time points. We analysed our data in three ways: firstly, to document trends from the first league season (1992/1993 for EPL; 1998/1999 for SPL) to present day; secondly, to document trends from 2005/2006 season to present day (to account for the shift brought by the 2005 Gambling Act); and thirdly, we focused on the 2016/2017 season. For the latter, we only looked at clubs who were competing in the EPL or SPL during the 2016/2017 season. Finally, we compared Poisson distributions parameterised by the sample mean of the frequency of gambling-related shirt sponsorships observed before 2005 to assess the likelihood that the frequencies observed after 2005 represent ‘chance’ findings, or are more likely to reflect a fundamental change in underling rates of shirt sponsorship in the EPLG.

## Findings

### First league season to present

**Figure 1:**
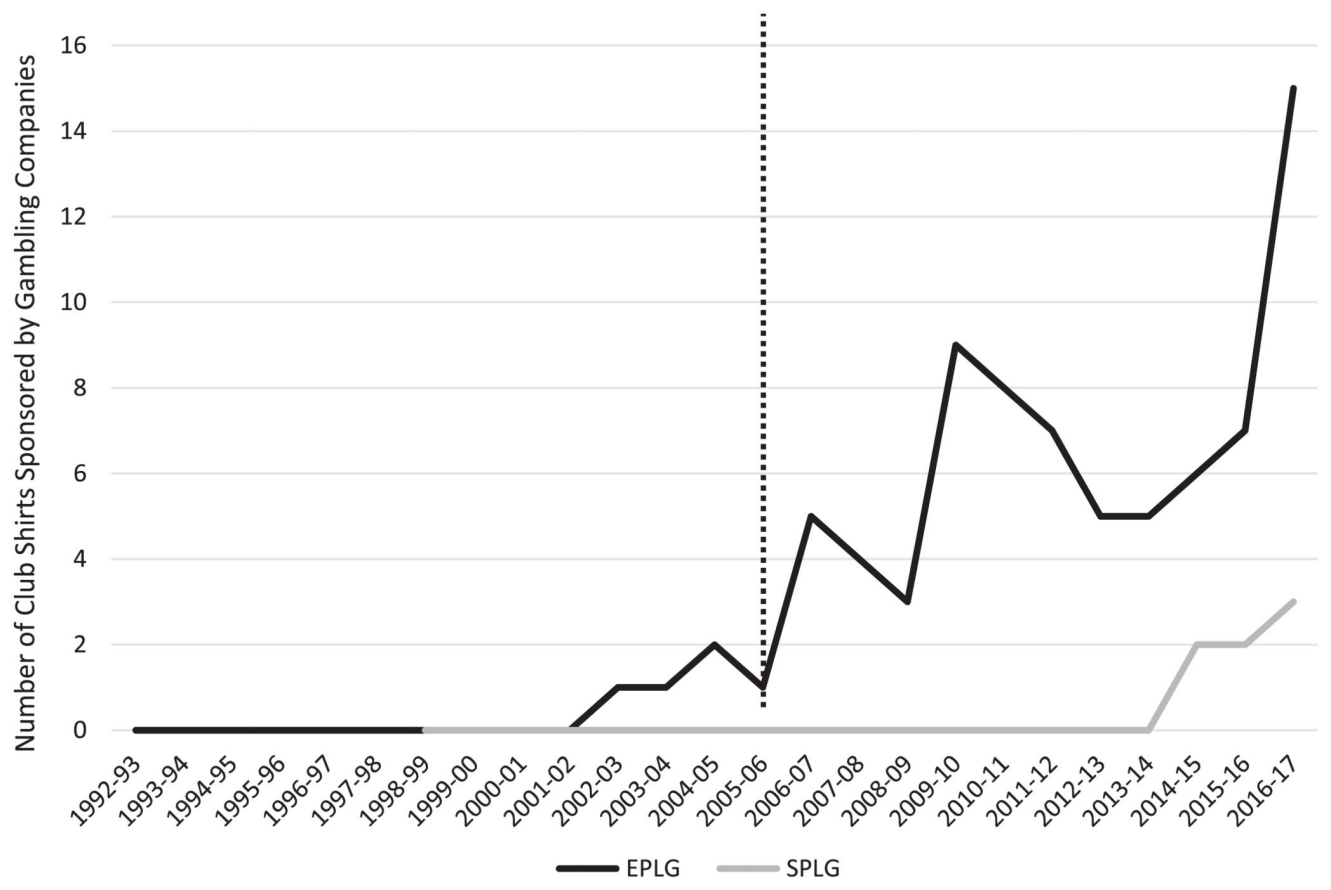
Comparison of current and former EPLG (*n* = 38) and SPLG (*n* = 19) receiving shirt sponsorship by gambling companies. Dotted line represents the introduction of the Gambling Act 2005.

We successfully identified the shirt sponsors of all 38 clubs who have participated in the EPLG and the 19 clubs in the SPLG, for all seasons since the inaugural season. The first gambling sponsor to appear on an EPLG shirt occurred in 2002/2003 season and in the 2014/2015 season in SPLG. The introduction of gambling companies as shirt sponsors in the SPL was 12 years behind the first EPL sponsorship deal. Figure 1 depicts the growth in shirt sponsorship in the two sets of clubs since the formation of each of the leagues. For each year, the range for EPLG members is 0–15 and 0–3 for SPLG members. The mean number of EPLG club shirts carrying gambling sponsorship since 1992/1993 was 3.16, compared to 0.37 in the SPLG since 1998/1999.

### 2005/2006 season to present

Since the 2005/2006 EPL and SPL seasons, an increase in gambling sponsorship can be observed. During this period, the range for EPLG members was 1–15 and 0–3 for SPLG members. Mean number of shirts carrying gambling sponsorship was 6.25 for EPLG clubs and 0.58 for SPLG clubs. Shirt sponsorship by a gambling company was dramatically more likely to occur amongst clubs who have been members of the EPLG than of the SPLG. The dotted line on Figure 1 indicates the introduction of the Gambling Act 2005. Of the documented instances of EPLG shirts sponsored by gambling companies, 95% (75/79) occurred after the introduction of this legislation.

### 2016/2017 season

**Figure 2:**
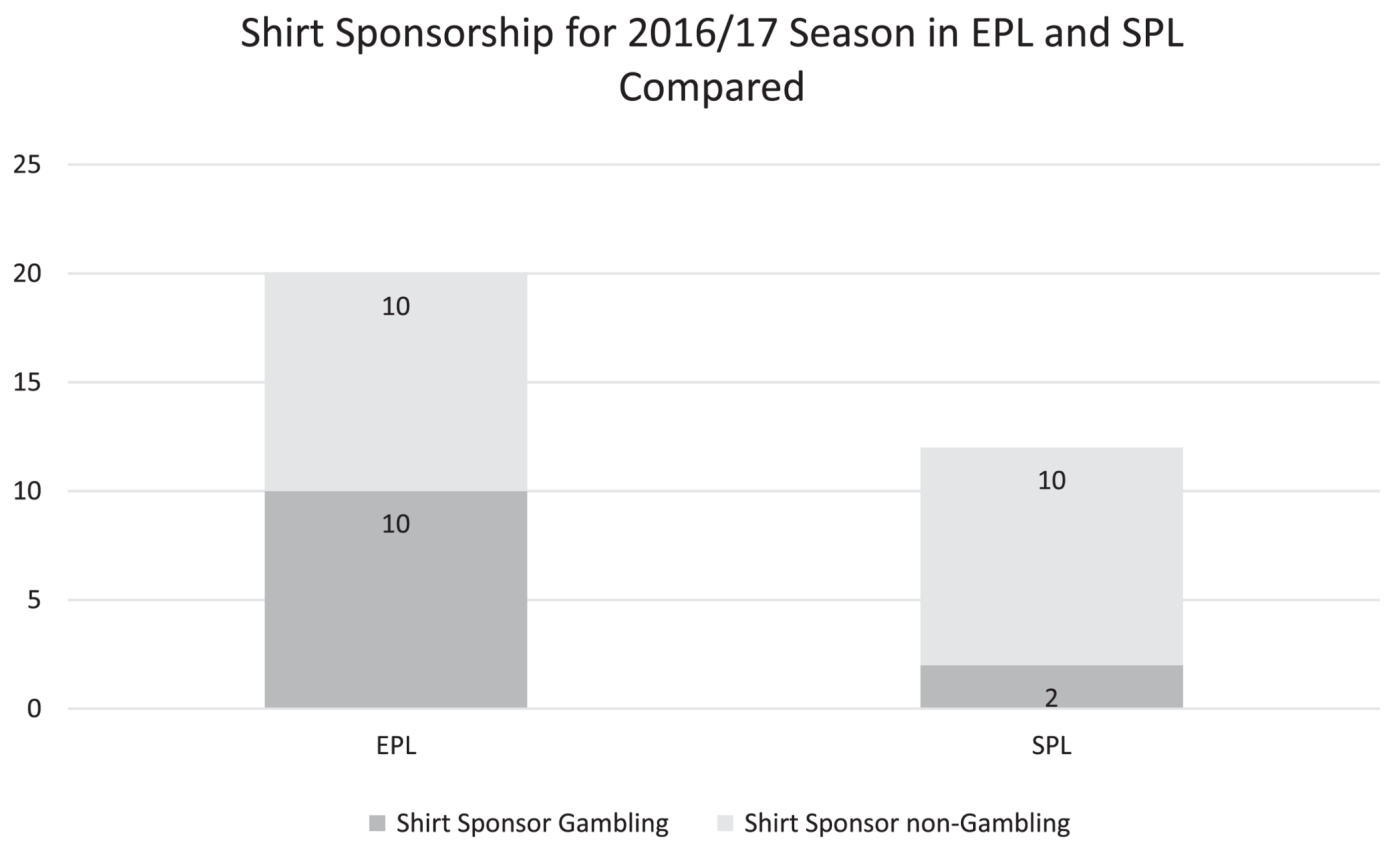
Comparison of current EPL and SPL clubs receiving shirt sponsorship by gambling companies in 2016/2017 season.

In the 2016/2017 EPL season 10 out of 20 (50%) *current league members* carry gambling sponsorship on their shirts. In contrast to this, such sponsorship arrangements are found in only 2 out of 12 (16.6%) of *current SPL members*. A visual representation of this can be found in Figure 2.

### Pre vs post 2005–2006 season

Before 2005, the mean number of shirts in the EPLG sponsored by gambling companies was 0.31. It was 6.25 for the post-2005 period. The proportion of values exceeding 6.25 expected from a Poisson distribution with a mean (lambda) value of 0.31 is exceedingly small (less than five per 100 million) and so we can reasonably conclude that there has been a change in the fundamentals of the determinants of sponsorship after the change in legislation.^38^ When the analysis was reversed, the likelihood of the values after 2005 being as low or lower than before 2005 was under two in 1000.^39^ This suggests that conditions operating in the two sponsorship environments are fundamentally different.

## Discussion

Our data suggest a pronounced increase in the presence of shirt sponsorship by gambling brands in the EPLG and a modest increase in the SPLG. Although we employ observational and not causal analysis, the correlation we observe in the EPLG is highly likely to be influenced by the Gambling Act 2005 for three reasons. First there is a clear causal mechanism as the Act relaxed gambling laws. Second, most of the increase we observe occurs after the Act’s introduction. Third, the magnitude of the increase was substantial. Other evidence has suggested that liberalizing gambling legislation in the UK has, historically, led to an increase in household spending on gambling.^40^ That gambling companies have responded to change in the legislative environment by seeking to increase their visibility through sponsoring teams, in the context of a likely rise in gambling participation, is commercially rational. Indeed, between the 2008 and 2009 (the year after the 2005 Act came into force) and 2015–2016 tax years, the UK gross gambling yield grew from £8.4bn to £13.6bn.^41^ This suggests that a key target for public health approaches to gambling in the UK is legislation.

The contrast between the EPL and SPL data is quite stark. Gambling companies have not sought shirt sponsorship opportunities in the SPLG in the same way as they have amongst the EPLG team. This is arguably due to the considerable difference in global reach: whereas EPL football enjoys a vast and global TV audience, SPL football attracts comparatively low levels of global coverage. From a phenomenological point of view, the world-wide EPL audiences are now in the position where 50% of the players they are watching play football carry the branding of gambling companies. So too do many of the advertising hoardings that surround the pitch, and the commercial breaks offer further reinforcement of the exhortation to wager. Many of the messages carried by these mediums encourage receivers to engage with social media and app-driven content, which when combined with smart phone technologies, make gambling even more accessible and imminent, and therefore potentially impulsive. This may reflect the fact that the UK market and social setting is not the limit for gambling organizations, as the literature on Africa we summarized earlier points out. The reach of the EPL makes the growth in gambling sponsorship on shirts a particularly concerning issue for *global* public health: shirt-borne adverts are now viewed with regularity across the globe, including in some of the poorest regions. Bet365, online gambling operator and sponsors of Stoke City’s shirt and stadium, is a good example. This company also operates using the same branding in Nigeria (http://www.bet365naija.com), as noted by Akanle and Kolade. In this instance, a domestic sponsorship deal supports a gambling company’s involvement in an overseas market that is characterized by poverty and funded in part by state benefits.^42^ The consequence of gambling liberalization in Britain, combined with the global broadcast strategies of the EPL, may therefore contribute to problematic gambling in low-income settings where fewer support systems are in place.

It should be noted that the difference between our findings for the EPLG and SPLG relates only to shirt sponsorship. For, while significantly less shirt sponsorship from gambling companies is happening in Scotland than in England, in other domains of comparison, there is less difference. For example, gambling companies sponsor the top four leagues in Scotland, as well as the league cup; in England, the same is true of the all three tiers of the English Football League (i.e. Championship down to League Two), but not in the EPL, which has not sold name rights to the league since 2016. All clubs competing in a gambling-sponsored league, whether they carry a gambling sponsor as the main shirt sponsor or not, receive money that is derived from this sponsorship. Furthermore, by sponsoring a league, gambling companies are able to insert their logos onto the upper-arm of team shirts, and into the graphics used in television coverage. While the English data suggest shirt sponsorship is a major vehicle for gambling companies’ commercial strategies, league sponsorship is common to both territories.

While some position gambling as a normal leisure activity, as an extension of sport – and for many this indeed is true – there are others for whom it can produce disordered behaviours that lead to declines in mental health, breakdowns in relationships, suicide and, ultimately, public cost.^43^Suggestions for regulating gambling sponsorship in sports have been made. Monaghan and colleagues draw together lessons from tobacco and alcohol studies to make policy recommendations aimed at minimizing the potential for harm in younger groups.^44^ Of particular note is their proposal to limit television adverts to times of the day when children and young people are less likely to view them. This seems particularly relevant in the current UK context, where gambling companies are free to air adverts during football matches at any time of the day.^45^ In the UK, regulations were put in place in 2007 to ensure that children’s replica shirts do not carry gambling sponsorship.^46^ While this was a welcome move, in the context of a football media environment saturated by gambling branding, it may well make little difference to the exposure young fans have to such brands (also, children tend to wear their own kits and look at adults’ kits). More recently, the English FA ended all sponsorship agreements with betting companies and the UK Labour Party has announced a commitment to ban gambling advertising on shirts, suggesting there is already an audience for further regulatory discussion and action.^47^

In addition to concerns about the vulnerability of children and young people to exposure to gambling sponsorship, it is known that certain medical treatments – most notably dopamine agonists such as levadopa for the treatment of Parkinson’s disease – can greatly increase the risk of disordered gambling behaviours developing.^48^ With rapid population ageing in most of the world, and rising medicalization of older population groups to treat Parkinson’s along with other morbidities associated with increasing longevity, greater visibility of gambling websites and services on football shirts could also have adverse consequences for those at risk of disordered gambling behaviours for this and other reasons.

The assumption that the EPL is more globally visible than the SPL, and that this influences the sponsorship dealings of gambling companies in UK football, is a limitation of our study, but generates a hypothesis worth testing. A second limitation is that we did not document the shirt sponsorship of all professional clubs in England and Scotland. This would have provided a more detailed account of shirt sponsorship and gambling in UK football. A further limitation is that we focused only on shirt sponsorship. Football clubs are increasingly turning to alternative sponsorship revenue-streams such as ‘club partners’. These ‘partners’ often include gambling organizations. Finally, the approach also fails to account for advertising hoardings and in-programme adverts, social media and app-driven content, all of which are utilized by gambling organizations as marketing spaces connected to football. These limitations all offer opportunities for future research in this important area.

With these limitations in mind, our study still makes an important contribution to the literature on sport and gambling. Little has been written about EPL and SPL relationships to gambling, and social science and public health analyses of such relationships in the UK are lagging behind the work done in contexts such as Australia and the US. As football, and especially the EPL, has such a vast global audience, understanding the consequences of clubs’ sponsorship deals is an important research agenda that has a potentially significant contribution to make to public health globally.

## Conclusion

The (often virtual and global) social setting that is the EPL is laden with the messages and branding of gambling companies. In the SPL, this is less true in terms of shirt sponsorship, but the branding of the leagues and league cup have provided gambling companies with an alternative means of raising their visibility and influencing the social setting. The close relationship between gambling companies and football in the UK, whether through shirt sponsorship or other media, arguably supports the normalization of gambling. While individual responsibility and control matter, the settings of the EPL and SPL are increasingly offering inducements to gamble. Given that we know that gambling can become disordered, public health academics, advocates and practitioners have a continued responsibility to raise and debate the issue of gambling’s relationship to sport.

The consequences of gambling disorders are well documented. Those afflicted often face significant personal and inter-personal harm. Societies and their governments also incur costs. The widespread association between the globally-visible EPL football clubs and the gambling industry raises substantial questions for football authorities and legislative authorities that have degrees of control over the relationship between football club shirt sponsorship (as well as other aspects of marketing strategies) and the gambling industry. In Scotland, the association is also growing: all four professional leagues are sponsored by a gambling company, and so too is the league cup. Given this apparent growth in visibility of gambling company branding and messaging in EPL and SPL contexts, public health in the UK should respond accordingly by pressing the issue with relevant public authorities, football governing bodies, broadcast and online rights holders, and individual clubs. For, all these groups have a responsibility to consider the ethical issues related to the environment that is created for their (global) supporters when they engage with football. Our findings suggest that legislators should revisit the impact that the Gambling Act 2005 has had on football sponsorship and its potential relationship to gambling-related harms.

## Notes

1 See Orford, “Gambling in Britain.”

2 Bernhard et al., “Gambling and Globalization,” 624.

3 See EPL, “Global Media Platform” and “Global Fan Base.”

4 See BBC, “Premier League Record Deal.”

5 Ahead of the 2013/2014 season, the Scottish Premier League was renamed the Scottish Professional Football League Premiership. For ease of writing, we have used ‘SPL’ to refer to both.

6 See Noel, “The Most Passionate Football Nation in Europe” and SPFL, “SPFL Increases Global TV Reach.”

7 See Murray, “£80 m TV deal.”

8 See APA, “Diagnostic and Statistical Manual of Mental Disorders” (3rd ed).

9 See APA, “Diagnostic and Statistical Manual of Mental Disorders” (5th ed).

10 Conversano et al., “Pathological Gambling.”

11 See Grant et al., “Medication Management of Pathological Gambling.”

12 Shaffer and Korn, “Gambling and Related Mental Disorders”, 201.

13 See Reith and Dobbie, “Beginning Gambling” and Reith, “Beyond Addiction or Compulsion.”

14 Reith and Dobbie, “Gambling Careers.”

15 See Grun and McKeigue, “Prevalence of Excessive Gambling.”

16 See Wardle, “British Gambling Prevalence Survey.”

17 See Wood and Griffiths, “A Qualitative Investigation Gambling” and Kalischuk et al., “Problem Gambling and Families.”

18 See Thorley et al., “Cards on the Table.”

19 See Maher et al., “Patterns of Sports Sponsorship.”

20 See Thomas et al., “Sports Betting Marketing.”

21 See Lindsay et al., “Eat, Drink and Gamble.”

22 See Hing et al., “Gambling Sponsorship of Sport.”

23 See Hing et al., “Adolescent Exposure to Gambling.”

24 See Bestman et al., “Children’s Implicit Recall.”

25 See Lamont et al., “Affective Response to Gambling.”

26 See McKelvey, “The Growth in Marketing.”

27 See Paul and Weinbach, “The Determinants of Betting.”

28 See Wann, “An Examination of Sport.”

29 See Gassmann et al., “Who Bets on Sports?”

30 See Jones, “Football, Alcohol and Gambling.”

31 See Vokes, “Arsenal in Bugamba.”

32 See Akanle and Taiwo Kolade, “European Football Clubs.”

33 See Akanle and Taiwo Kolade, “Football Betting in Nigeria.”

34 See Akanle and Taiwo Kolade, “European Football Clubs.”

35 See Bourdieu, “Economy of Symbolic Goods.”

36 See European Football Statistics, “Nottingham Forest FC.”

37 See Fitba Stats, “Hibernian.”

38 This analysis can be performed using the open source and free statistical package, *R*, by typing ppois(*q* = 6.25, lambda = 0.31, lower.tail = *F*), which calculates the proportion of values that would be expected to be above 6.25 generated from a Poisson distribution with a lambda (mean) value of 0.31. The precise output produced in *R* is 4.164439e-08. Alternative model specifications, such as the Negative Binomial model, could also be used, and the effect of incorporating parameter uncertainty due to sampling error could also be explored, but are very unlikely to change the substantive conclusions drawn about whether the frequency of shirt sponsorship observed after the change in legislation are fundamentally higher than those observed before.

39 This analysis can be performed using the open source and free statistical package, *R*, by typing ppois(*q* = 0.31, lambda = 6.25, lower.tail = *T*), which in this case calculates the proportion of values that would be expected to be below 0.31 generated from a Poisson distribution with a lambda (mean) value of 6.26. The precise output produced in *R* is 0.001930454. Alternative model specifications, such as the Negative Binomial model, could also be used, and the effect of incorporating parameter uncertainty due to sampling error could also be explored, but are very unlikely to change the substantive conclusions drawn about whether the frequency of shirt sponsorship observed before the change in legislation are fundamentally lower than those observed afterwards.

40 See Grun and McKeigue, “Prevalence of Excessive Gambling” and Wardle et al., “British Gambling Prevalence Survey.”

41 See PGRI, “UK Gambling Industry Data Report.”

42 See Akanle and Taiwo Kolade, “European Football Clubs.”

43 See Abt et al., “Gambling: The misunderstood sport,” Cowlishaw and Kessler, “Problem Gambling in the UK,” Pfuhlmann and Schmidtke, “Pathological Gambling, Suicidal Behavior” and Thorley et al., “Cards on the Table.”

44 See Monaghan et al., “Impact of Gambling Advertisements.”

45 See Jones, “Football, Alcohol and Gambling.”

46 See Temko, “New Code to Restrict.”

47 See BBC, “Labour: Scrap Betting Ads” and FA “Football Association Board.”

48 See Dodd et al., “Pathological Gambling, Parkinson’s Disease.”
